# Identification of Enzymes Oxidizing the Tyrosine Kinase Inhibitor Cabozantinib: Cabozantinib Is Predominantly Oxidized by CYP3A4 and Its Oxidation Is Stimulated by cyt b_5_ Activity

**DOI:** 10.3390/biomedicines8120547

**Published:** 2020-11-28

**Authors:** Radek Indra, Katarína Vavrová, Petr Pompach, Zbyněk Heger, Petr Hodek

**Affiliations:** 1 Department of Biochemistry, Faculty of Science, Charles University, Albertov 6, 12800 Prague 2, Czech Republic; katarina.vavrova@natur.cuni.cz (K.V.); petrpompach@gmail.com (P.P.); petr.hodek@natur.cuni.cz (P.H.); 2 Department of Chemistry and Biochemistry, Mendel University in Brno, Zemedelska 1, 61300 Brno, Czech Republic; zbynek.heger@mendelu.cz; 3 Central European Institute of Technology, Brno University of Technology, Purkynova 656/123, 61200 Brno, Czech Republic

**Keywords:** cabozantinib, cytochrome P450, tyrosine kinase inhibitor, cytochrome b_5_

## Abstract

Herein, the in vitro metabolism of tyrosine kinase inhibitor cabozantinib, the drug used for the treatment of metastatic medullary thyroid cancer and advanced renal cell carcinoma, was studied using hepatic microsomal samples of different human donors, human recombinant cytochromes P450 (CYPs), flavin-containing mono-oxygenases (FMOs) and aldehyde oxidase. After incubation with human microsomes, three metabolites, namely cabozantinib *N*-oxide, desmethyl cabozantinib and monohydroxy cabozantinib, were detected. Significant correlations were found between CYP3A4 activity and generation of all metabolites. The privileged role of CYP3A4 was further confirmed by examining the effect of CYP inhibitors and by human recombinant enzymes. Only four of all tested human recombinant cytochrome P450 were able to oxidize cabozantinib, and CYP3A4 exhibited the most efficient activity. Importantly, cytochrome b_5_ (cyt b_5_) stimulates the CYP3A4-catalyzed formation of cabozantinib metabolites. In addition, cyt b_5_ also stimulates the activity of CYP3A5, whereas two other enzymes, CYP1A1 and 1B1, were not affected by cyt b_5_. Since CYP3A4 exhibits high expression in the human liver and was found to be the most efficient enzyme in cabozantinib oxidation, we examined the kinetics of this oxidation. The present study provides substantial insights into the metabolism of cabozantinib and brings novel findings related to cabozantinib pharmacokinetics towards possible utilization in personalized medicine.

## 1. Introduction

In general, differences in drug absorption, distribution, metabolism and excretion result in interindividual variations in the pharmacokinetics of a drug, if patients are administered a uniform dose of the drug [1]. Among patients with benign diseases treated with common drugs, relatively small differences in drug pharmacokinetics including metabolism are observed because of the wide therapeutic windows. However, in cancer chemotherapy, serious clinical consequences may occur from small alterations in drug metabolism affecting the drug pharmacokinetics [2].

Cabozantinib (*N*′1-{4-[(6,7-dimethoxyquinolin-4-yl)oxy]phenyl}-*N*1-(4-fluorophenyl)cyclopropane-1,1-dicarboxamide) exhibits substantial antitumor activities in patients with multiple cancer types: medullary thyroid cancer [3], breast cancer [4,5], hepatocellular carcinoma [6,7], non-small cell lung cancer [8,9], prostate cancer [10,11], renal-cell carcinoma [12,13] and pancreatic cancer [14]. Pursuant to these studies, cabozantinib was approved as COMETRIQ^®^ for treatment of metastatic medullary thyroid cancer in 2012 and as CABOMETYX™ for patients with advanced renal cell carcinoma in 2016. Moreover, cabozantinib as CABOMETYX™ was approved in 2019 for hepatocellular carcinoma patients who have been previously treated with sorafenib. It acts as a tyrosine kinase inhibitor (TKI), affecting vascular endothelial growth factor receptor 2 (VEGFR-2) and hepatocyte growth factor receptor MET [15,16,17,18]. It also inhibits several other kinases, including KIT, RET and AXL [15]. Therefore, cabozantinib is considered a multiple TKI, affecting tumor angiogenesis, invasion and metastasis. Although cabozantinib is well tolerated, its administration is associated with frequent treatment-limiting adverse effects, and dose reductions are commonplace. The most frequently reported adverse effects in patients with renal-cell carcinoma are diarrhea, palmar–plantar erythrodysesthesia syndrome, fatigue and hypertension [4,7,19].

Cabozantinib is efficiently bound by proteins in human plasma (≥99.7%) [20]. Recent work with healthy volunteers revealed that cabozantinib was eliminated primarily in feces and extensively metabolized up to 17 metabolites identified by liquid chromatography-tandem mass spectrometry [18]. These metabolites were represented by hydroxy-derivates, amide cleavage products, glucuronide conjugates and sulfates. Thus, enzymes of phase I and also phase II participate in cabozantinib metabolism in the human body. Because of limited information, which indicates lower efficiency of individual cabozantinib metabolites [18], knowledge of the cabozantinib metabolic pathway is crucial for the improvement of treatment protocols and prognostic accuracy.

In the present study, we investigated the in vitro metabolism of cabozantinib in cell-free systems. We used human hepatic subcellular systems (microsomes) containing enzymes that are responsible for the first-pass metabolism of drugs. In addition, individual recombinant human cytochromes P450 (CYPs), flavin-containing mono-oxygenases (FMOs) and aldehyde oxidase (AO) were employed to identify enzymes capable of metabolizing cabozantinib. Since CYP3A4 was identified as the major enzyme metabolizing cabozantinib in human liver, the mechanism of CYP3A4-catalyzed oxidation was characterized in detail.

## 2. Materials and Methods

### 2.1. Chemicals and Materials

Cabozantinib was from MedChemExpress (Monmouth Junction, NJ, USA). Standard of cabozantinib *N*-oxide was purchased from Toronto Research Chemicals (Toronto, Canada). Ketoconazole, NADPH and other chemicals were all purchased from Merck (Darnstadt, Germany). The purity of all chemicals met the standards of American Chemical Society, unless otherwise noted. Male human hepatic microsomes (pooled sample) (sample LOT: 3043885) were from Gentest Corp. (Woburn, MA, USA). Microsomes from livers of twelve human donors were obtained from Gentest Corp. (Woburn, MA, USA) (Lot. no. HG43-1, HG103, HG74, HG93, HG24, HG27, HG23, HG32, HK31, HK34, HG03 and HG42). Each human microsomal sample was characterized for CYP, FMO, total protein contents and specific CYP activities by Gentest Corp. and reanalyzed by us by assays described in the protocols of Gentest Corp. Our data were similar to those reported by Gentest Corp. (Table 1). Human recombinant enzymes were used in the forms of Supersomes™ and obtained from Corning (Corning, NY, USA). In Supersomes™, microsomal fractions were isolated from insect cells that were transfected with baculovirus constructs containing cDNA of human CYP enzymes (CYP1A1/2, 1B1, 2A6, 2B6, 2C8/9/18/19/, 2D6, 2E1, 3A4). The Supersomes™ also express NADPH:CYP oxidoreductase (POR). However, because they are microsomes (particles of broken endoplasmic reticulum), other enzymes (proteins) of the endoplasmic reticulum membrane (like NADH:cytochrome b_5_ reductase and cytochrome b_5_) are also expressed at basal levels in these Supersomes™. We also utilized Supersomes™, which overexpressed cytochrome b_5_, in a molar ratio of CYP to cytochrome b_5_ of 1 to 5. In Supersomes™, where cytochrome b_5_ was not overexpressed (see above), pure cytochrome b_5_ was added to reach a molar ratio of CYP to cytochrome b_5_ of 1 to 5. Reconstitution of Supersomes™ with purified cytochrome b_5_ was performed as described previously [21,22,23]. Human recombinant FMOs and AO were also used in the forms of Supersomes™ (Gentest Corp., Woburn, MA, USA). These microsomal fractions were isolated from insect cells that are transfected with baculovirus constructs containing cDNA of human FMO1, FMO3, FMO5 or AO enzymes.

### 2.2. Oxidation of Cabozantinib by Hepatic microsomes and Human Recombinant Enzymes

Unless stated otherwise, incubation mixtures used to study cabozantinib metabolism contained the following in a final volume of 500 μL for incubations containing hepatic microsomes and 250 μL for incubations with recombinant enzymes (Supersomes™): 100 mM potassium phosphate buffer (pH 7.4); 1 mM NADPH; human hepatic microsomes (0.25 mg protein) or human recombinant CYPs, FMOs or AO in Supersomes™ (25 pmol); and 50 µM cabozantinib dissolved in 5 (respectively 2.5) µL dimethyl sulfoxide. The reaction was initiated by adding NADPH. In control incubations, either microsomes or CYP (FMO, AO) or NADPH were omitted. Incubations were performed at 37 °C for 20 min in open plastic Eppendorf tubes; cabozantinib oxidation was linear up to 30 min of incubation. The reaction was stopped by extraction with ethyl acetate (2 × 1 mL). Extracts were evaporated and dissolved in 50 µL methanol, and high-performance liquid chromatography (HPLC) analysis was used to separate cabozantinib and its metabolites. HPLC conditions were as follows: Nucleosil^®^ EC 100-5 C18 reverse-phase column (150 × 4.6 mm, Macherey Nagel, Duren, Germany); the eluent was 5 mM ammonium acetate in water (pH 5) containing 60% acetonitrile with a flow rate of 1 mL/min; injection was 10 µL; and detection was at 254 nm. Cabozantinib metabolites separated by HPLC were characterized by mass spectroscopy (see further details below). The recoveries of cabozantinib metabolites were approximately 95%.

### 2.3. Identification of Cabozantinib Metabolites by Mass Spectrometry

Cabozantinib metabolites were identified by liquid chromatography coupled with Q-ToF mass spectrometer. Samples were re-suspended in 50 µL of methanol. Ten microliters was injected via autosampler (Ultimate 3000 UHPLC system, Thermo Fisher Scientific, Waltham, MA, USA) on reverse-phase C18 column (150 × 4.6 mm, Macherey Nagel) heated at 37 °C. Cabozantinib metabolites were eluted isocratically by 5 mM ammonium acetate in water (pH 5) containing 60% acetonitrile solvent at a flow rate of 0.6 mL/min and measured by Q-ToF mass spectrometer (maXis Plus, Bruker Daltonics, Bremen, Germany) operating in positive data-dependent mode. Mass range was 50–700 *m*/*z*, number of precursors was 3 and a spectral rate was set at 5.00 Hz. Mass spectrometer was externally calibrated using NaTFA. Data were processed by DataAnalysis 4.3 software (Bruker Daltonics).

### 2.4. Inhibition Study

Inhibition studies in pooled human liver microsomes were conducted with six inhibitors. The inhibitors employed were as follows: α-Naphthoflavone (α-NF), which inhibits CYP1A1 and 1A2; sulfaphenazole, which inhibits CYP2C; quinidine, which inhibits CYP2D; diethyldithiocarbamate (DDTC), which inhibits CYP2E1 and CYP2A; ketoconazole, which inhibits CYP3A; and CYP3cide, which inhibits CYP3A4. The IC_50_ values for employed inhibitors were determined by the procedure described previously [24]. For experiments, inhibitors were dissolved in 5 µL methanol and incubated at 37 °C for 10 min with 50 µM cabozantinib followed by the addition of NADPH. Then, mixtures were incubated for a further 20 min at 37 °C. Formation of cabozantinib metabolites was analyzed by HPLC as described above.

### 2.5. Contributions of CYP Enzymes to Formation of Cabozantinib Metabolites in Human Livers

In order to calculate the contributions of individual CYPs to the formation of individual cabozantinib metabolites in human liver, we measured the velocities of their formation by the Supersomal CYP enzyme systems containing cyt b_5_ and combined these velocities with data on the average expression levels of individual CYPs in human liver derived from [25,26,27]. Specifically, the contributions of each CYP to cabozantinib metabolite formation in liver were calculated by dividing the relative metabolite-forming activity of each CYP (rate of formation multiplied by amounts of this CYP in human liver) by the total relative activities of all metabolite-forming CYPs.

### 2.6. Statistical Analysis

Data are expressed as mean ± SD. Data were analyzed using GraphPad Prism 7 (San Diego, CA, USA) using ANOVA with post-hoc Tukey HSD Test. All *p*-values are two-tailed and considered significant at the 0.05 level. Statistical associations between CYP- and FMO-linked catalytic activities in human hepatic microsomal samples and amounts of cabozantinib metabolites were determined by linear regression using Statistical Analysis System software version 6.12. Correlation coefficients (r) were based on a sample size of twelve for human microsomes. All *p*-values are two-tailed and considered significant at the 0.05 level.

## 3. Results

### 3.1. Oxidation of Cabozantinib by Human Hepatic Microsomes and Correlation of CYPs Activities with Cabozantinib Oxidation

**Figure 1:**
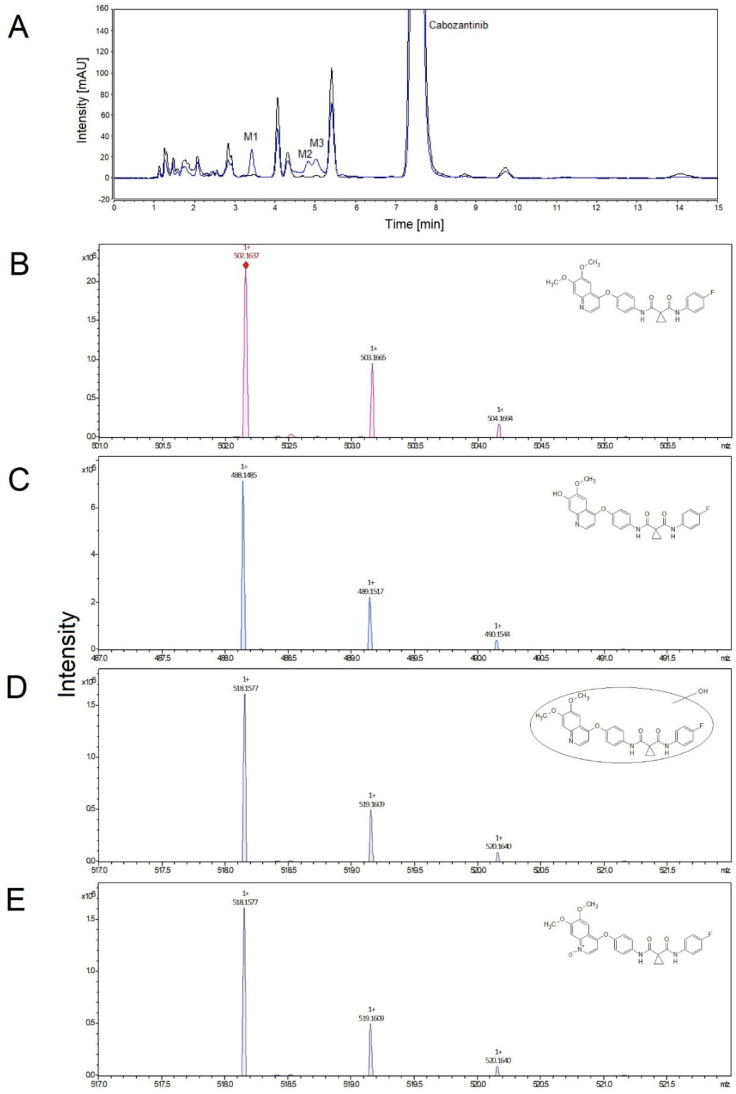
Representative HPLC chromatogram of cabozantinib metabolites formed in the presence of NADPH (blue line) and without NADPH (control, black line). M1—monohydroxy cabozantinib; M2—desmethyl cabozantinib; M3—cabozantinib *N*-oxide (**A**). Detailed mass spectrum and structure (insert) of cabozantinib (**B**), desmethyl cabozantinib (**C**), monohydroxy cabozantinib (**D**) and cabozantinib *N*-oxide (**E**).

First, we investigated the function of the human hepatic microsomal system, which contains biotransformation enzymes, from individual donors to catalyze the oxidation of cabozantinib. All hepatic microsomes oxidized cabozantinib to three metabolites that were separated by HPLC and identified by mass spectroscopy. In the case of cabozantinib *N*-oxide, the structure was confirmed by analyzing the standard of this metabolite. The formation of cabozantinib metabolites was dependent on NADPH, and without it, no oxidation of cabozantinib was detected (Figure 1). The predominant metabolite was identified as cabozantinib *N*-oxide (M_3_). Two other metabolites were identified as monohydroxy cabozantinib (M_1_) and desmethyl cabozantinib (M_2_).

The correlation analysis between enzyme activities of different CYPs in single-donor microsomes and the amounts of individual cabozantinib metabolites formed in each microsomal sample (Table 1) were used to examine the role of specific human CYPs in their generation. The highest correlation for all three metabolites was found with testosterone-6β-hydroxylation (a marker for CYP3A4): cabozantinib *N*-oxide (r = 0.947; *p* < 0.001), monohydroxy cabozantinib (r = 0.918; *p* < 0.001) and desmethyl cabozantinib (r = 0.935; *p* < 0.001). Significant correlations were also found between individual metabolites and activities of three other cytochromes P450 (CYP2A6, 2B6 and 2C8; Table 2). These results indicate that these four enzymes might be responsible for the formation of cabozantinib metabolites in human liver. However, there are high cross-correlations between testosterone-6β-hydroxylation and coumarine-7-hydroxylase (marker of CYP2A6; r = 0.786; *p* < 0.01), testosterone-6β-hydroxylation and (S)-mephenytoin-*N*-demethylase (marker of CYP2B6; r = 0.823; *p* < 0.01), testosterone-6β-hydroxylation and paclitaxel-6α-hydroxylase (marker of CYP2C8; r = 0.751; *p* < 0.01) and (S)-mephenytoin-*N*-demethylase and paclitaxel-6α-hydroxylase (r = 0.930; *p* < 0.001) within these liver microsomes samples. To clarify these correlations, multivariate analysis was used to investigate the dependence of metabolite formation on these CYP activities. Activities of CYP2A6, -2B6 and -2C8 in each microsomal sample were combined in pairs with the activities of CYP3A4 to see if any combination of the activities led to an improvement in the correlation with CYP3A4. We can conclude that CYP3A4 is in fact the enzyme responsible for cabozantinib oxidation.

### 3.2. The Effect of CYP Enzyme Inhibitors on Cabozantinib Oxidation in Human Liver Microsomes

The importance of CYP3A4 in cabozantinib oxidation was confirmed by inhibition studies with human pooled liver microsomes from 21 donors. CYP-specific inhibitors α-Naphthoflavone (α-NF, CYP1A), sulfaphenazole (CYP2C) and diethyldithiocarbamate (DDTC, CYP2A and CYP2E1) were inefficient in affecting the cabozantinib oxidation. Quinidine (inhibiting CYP2D) was able to inhibit formation of metabolites; however, IC_50_ was not attained. Thus, only inhibitors of CYP3A, namely ketoconazole and CYP3cide, were efficient in inhibiting the oxidation of cabozantinib. Both inhibitors were so efficient that, even in the lowest concentration, the formation of metabolites was inhibited by more than 50%, so the values of IC_50_ are < 0.01 µM. The only exception was monohydroxy cabozantinib, which exhibited IC_50_ of 1.12 µM for CYP3cide. Due to the higher efficiency of ketoconazole than CYP3cide, it is possible to suppose an oxidative role not only of CYP3A4 but also of CYP3A5 during cabozantinib oxidation in human liver microsomes. However, the role of CYP3A5 in this phenomenon seems to be minor in comparison to CYP3A4.

### 3.3. Oxidation of Cabozantinib by Recombinant Human Cytochromes P450

**Figure 2:**
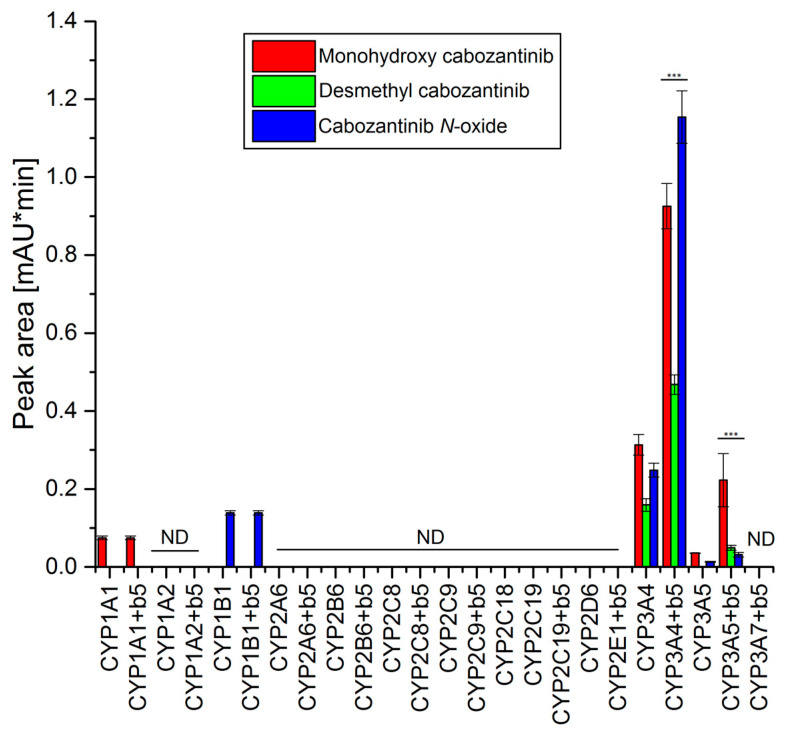
Oxidation of cabozantinib by human recombinant CYPs. Value represents mean ± SD of three independent in vitro incubations (*n* = 3). *** *p* < 0.001 significant differences between the formation of individual metabolites by CYP enzymes with and without cytochrome b_5_ (b_5_). ND—not detected.

**Figure 3:**
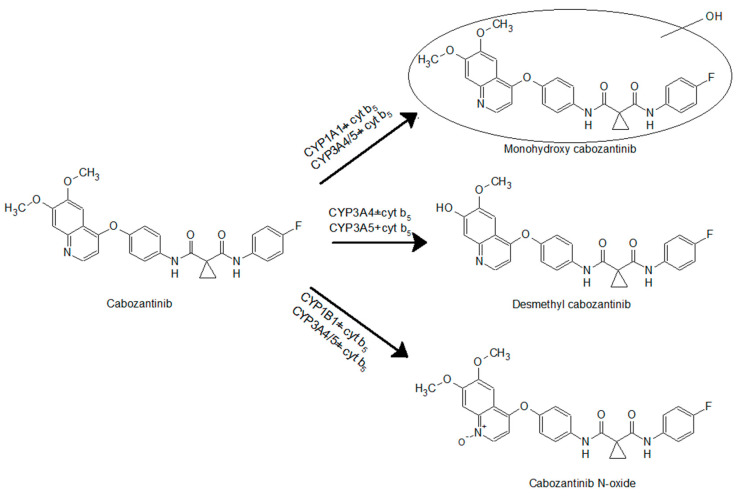
Cabozantinib oxidation by cytochrome P450s.

The use of recombinant CYP enzymes expressed in Supersomes™ was another approach to examine the ability of individual human CYP enzymes to oxidize cabozantinib. Of several tested CYPs, four cytochromes P450 were capable of oxidizing cabozantinib under the experimental conditions (Figure 2). CYP3A4, the most prominent enzyme oxidizing cabozantinib, generated three metabolites. Cyt b_5_, the heme protein participating in several functions of the CYP–mono-oxygenase system [21,28,29,30,31,32], exhibits a stimulating effect on all three metabolites (Figure 2). The use of Supersomes™ with co-expression of cyt b_5_ and CYP3A4 increased cabozantinib oxidation more than 2.9-fold. The greatest stimulatory effect of cyt b_5_ was found in the formation of cabozantinib *N*-oxide (4.6-fold; *p* < 0.001). Cyt b_5_ also stimulates cabozantinib oxidation by CYP3A5. Thus, desmethyl cabozantinib was detected only in its presence (Figure 2). CYP1A1 and CYP1B1 each generate one metabolite, and their activity was not influenced by cyt b_5_. CYP1A1 formed monohydroxy cabozantinib, while desmethyl cabozantinib was attributed to CYP1B1 (Figure 2). The proposed scheme of cabozantinib oxidation by human recombinant CYPs is shown in Figure 3.

Based on the results assigning the velocities of cabozantinib oxidation to individual metabolites in experimental systems containing recombinant CYP enzymes in Supersomes™ (Figure 2) and the relative amounts of CYP enzymes expressed in human liver [25,26,27], the contributions of individual CYPs to these reactions in human livers were evaluated. For these calculations, we took the presence of cytochrome b_5_ into account. The highest contribution to formations of all three metabolites was attributed to CYP3A4. Other CYPs contribute less than 3% to the formation of monohydroxy cabozantinib and even less than 1% to the formation of desmethyl cabozantinib and cabozantinib *N*-oxide.

**Figure 4:**
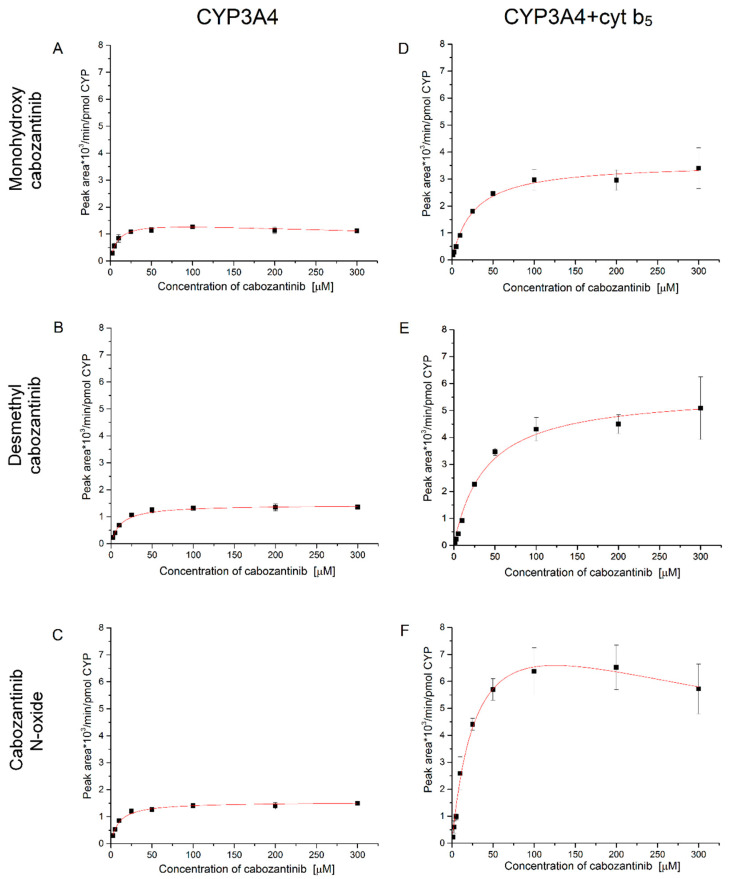
Enzyme kinetics of cabozantinib oxidation by CYP3A4 (**A**–**C**) and CYP3A4 in the presence of cytochrome b_5_ (**D**–**F**) to monohydroxy cabozantinib (**A**,**D**), desmethyl cabozantinib (**B**,**E**) and cabozantinib *N*-oxide (**C**,**F**). Values represent means ± SD of three independent in vitro incubations (*n* = 3).

Based on the obtained results, it is clear that cabozantinib is primarily oxidized by CYP3A4. Therefore we further investigated the kinetics of the CYP3A4-mediated cabozantinib oxidation (Figure 4 and Table 3). The presence and absence of cyt b_5_ was considered. In the case of CYP3A4, the metabolites desmethyl cabozantinib and cabozantinib *N*-oxide exhibit hyperbolic kinetics (Figure 4B,C). The third metabolite, monohydroxy cabozantinib, is prone to substrate inhibition (Figure 4A). When cyt b_5_ is present in the incubations, the rate of cabozantinib oxidation and the kinetics are affected. The cyt b_5_ increases the rate of cabozantinib oxidation, and a higher concentration of cabozantinib is required to attain half-maximal velocity. The K_0.5_ values triple with cytochrome b_5_, which indicates that allosteric effects may affect catalysis. The allosteric effects are also supported by the fact that the formation of monohydroxy cabozantinib exhibits hyperbolic kinetics, although without cyt b_5_, it exhibits substrate inhibition (Figure 4A,D). The formation of cabozantinib *N*-oxide is also affected by cyt b_5_ and is prone to substrate inhibition in its presence (Figure 4F). Desmethyl cabozantinib exhibits hyperbolic kinetics regardless of cytochrome b_5_ (Figure 4B,E). Thus, cyt b_5_ not only stimulates the cabozantinib oxidation by CYP3A4 but also affects the affinity of this enzyme.

### 3.4. Oxidation of Cabozantinib by Recombinant Human Flavin-Containing Mono-oxygenases and Aldehyde Oxidase

The potential of aldehyde oxidase and flavin-containing mono-oxygenases to oxidize cabozantinib was studied with human recombinant enzymes. Aldehyde oxidase and all three tested flavin-containing mono-oxygenases (FMO 1, 3 and 5) were ineffective in cabozantinib oxidation (data not shown).

## 4. Discussion

In the present study, we utilized several approaches to identify individual CYP enzymes oxidizing cabozantinib. Ketoconazole, an inhibitor of CYP3A [33,34], and CYP3cide, a specific inhibitor of CYP3A4 [35], were highly efficient in the inhibition of cabozantinib oxidation. Inhibitors of other CYPs, namely α-NF, sulfaphenazole and DDTC, showed no inhibitory effects or, in the case of quinidine, the value of IC_50_ was not reached. However, it is important to point out that results found with inhibitors are sometimes difficult to interpret because inhibitors can act more efficiently with one enzyme substrate than another and may influence multiple forms of cytochrome P450. Therefore, we employed additional experimental approaches: (i) correlation analysis between the CYP- and FMO-catalytic activities in each microsomal sample with the amounts of individual metabolites formed by the same microsomes; and (ii) analysis of the oxidation of cabozantinib by human recombinant CYPs, FMOs and AO. In the human hepatic microsomal systems, CYP3A4 was determined as the most important enzyme in the formations of cabozantinib metabolites based on correlation analysis. Human recombinant CYP3A4 expressed in Supersomes™ was also the most effective enzyme responsible for the formation of cabozantinib metabolites. The significant role of CYP3A4 in the oxidation of cabozantinib was proposed and indicated by others [36,37]. Many other TKIs are also known substrates of CYP3A4 [31,38,39,40,41]. All these data, and the fact that CYP3A4 is the predominant form of cytochrome P450 in human liver [25,42], indicate the privileged role of this enzyme in the oxidation of TKIs including cabozantinib.

The oxidation of cabozantinib by CYP3A4 is stimulated by cytochrome b_5_. The stimulatory effect of cyt b_5_ on CYP3A4-mediated oxidation is known for endogenous [28,43] as well as exogenous substrates [30,43,44,45,46], including TKIs [31]. However, the catalytic activity of CYP3A4 shows large interindividual variability depending on many factors such as modulation of CYP3A4 expression by xenobiotics and the diet or genetic polymorphism. Polymorphism in CYP3A4 genes has a great impact on enzymatic activities, thereby influencing the metabolism and elimination of drugs [42,47]. The effect of CYP3A4 polymorphism on the oxidation of cabozantinib to cabozantinib *N*-oxide was described recently [37]. Cytochrome b_5_ also exhibits interindividual variability. Protein and mRNA contents show variations with 11- and 6-fold range, respectively. However, the content of cyt b5 mRNA does not strongly correlate with that of cyt b_5_ protein [48]. Other studies also reported variability in cyt b_5_ protein and mRNA [49,50]. Correlation between cyt b_5_ and CYP3A4 activity was found [48]. Further, single nucleotide polymorphisms in cyt b_5_ were associated with very low activity and protein expression [51]. All these factors can contribute to interindividual differences in the CYP3A4-mediated pharmacokinetic profiles of cabozantinib.

Cabozantinib showed generally broader and more potent kinase inhibition compared to its metabolites. Estimated IC_50_ of cabozantinib for MET, RET and VEGFR2 are 2, 8 and 14 nM, respectively. Cabozantinib *N*-oxide activities against these kinases are 190, >1000 and 140 nM, respectively [18]. Effectiveness of cabozantinib might thus be significantly reduced by CYP3A4-mediated oxidation in enterocytes and liver cells. This can lead to different anticancer activity and/or adverse effects after cabozantinib treatment. Cabozantinib could cause many side effects, which require subsequent dose modification [52]. To better understand the forming of metabolites, kinetics of cabozantinib oxidation by CYP3A4, the most efficient enzyme oxidizing cabozantinib, was analyzed. All metabolites formed by CYP3A4 exhibit K_0.5_ from 9 to 12 µM and very similar V_Max_. Addition of cyt b_5_ into the incubation mixture causes an increase in these parameters. Particularly, K_0.5_ is approximately three times higher in the presence of cyt b_5_. Nevertheless, the change of V_Max_ is up to 6.5-fold higher. These results indicate that cyt b_5_ affects the affinity of CYP3A4. Further evidence for the influencing of affinity comes from changing hyperbolic kinetics into substrate inhibition in the case of cabozantinib *N*-oxide or vice versa in the case of desmethyl cabozantinib. Substrate inhibition for the formation of cabozantinib *N*-oxide was recently observed with many isoforms of CYP3A4 in the presence of cyt b_5_ [37]. Another previously published study claimed that cabozantinib is not the only substrate of CYP3A4 but also a direct inhibitor of this enzyme with a IC_50_ value 272 µM (midazolam 1′-hydroxylase), while cabozantinib did not inhibit CYP3A4-mediated testosterone 6β-hydroxylation [18]. Our results confirm the ability of cabozantinib to inhibit CYP3A4; however, the IC_50_ is higher than the value presented in the above-mentioned study. With respect to plasma concentration of cabozantinib in patients, it is unexpected for CYP3A4 inhibition by cabozantinib to be observed. Plasma concentration of cabozantinib in metastatic renal cell carcinoma patients has a mean of 973 ± 501 ng/mL. The range of measured concentrations was 203 to 2100 ng/mL [53]. In patients with thyroid cancers, a therapeutic window between 500–1500 ng/mL has been proposed for cabozantinib trough levels to ensure an acceptable efficacy/toxicity balance [54]. Metabolites formed by CYP3A4 exhibit K_0.5_ near 10 µM, thus only at three times higher concentration than is observed in patients. Because of the lower efficiency of cabozantinib metabolites, extensive metabolizers may require higher doses, and inducers and inhibitors of CYP3A4 should be avoided or dose modifications should be considered during cabozantinib treatment.

## 5. Conclusions

Cabozantinib *N*-oxide, desmethyl cabozantinib and monohydroxy cabozantinib were identified as major oxidation products of cabozantinib, the drug used for the treatment of metastatic medullary thyroid cancer and advanced renal cell carcinoma. CYP3A4 was identified as a prominent cytochrome P450 forming cabozantinib metabolites, which is responsible for nearly all cabozantinib oxidation in the human liver. The oxidation of cabozantinib by CYP3A4 is stimulated by cyt b_5_ and may be prone to substrate inhibition. Therefore, the presence of CYP3A4 modulators should be considered during cabozantinib treatment.

## Figures and Tables

**Table 1:** CYP- and FMO-dependent catalytic activities and amounts of cabozantinib metabolites in samples of human liver microsomes.

	Total CYPs ^a^	POR ^b^	Cyt b_5_^c^	CYP1A2 ^d^	CYP2A6 ^d^	CYP2B6 ^d^	CYP2C8 ^d^	CYP2C9 ^d^	CYP2C19 ^d^	CYP2D6 ^d^	CYP2E1 ^d^	CYP3A4 ^d^	CYP4A11 ^d^	FMO ^d^	M1 ^e^	M2 ^e^	M3 ^e^
HG03	290	450	380	170	2000	51	200	1700	44	110	1800	6100	1600		0.287	0.335	1.672
HG103	340	210	790	310	440	7.2	39	2300	23	65	1100	2200	1600	1400	0.109	0.144	0.644
HG24	260	260	550	1700	1500	35	190	3000	41	-	2300	4000	1800	1500	0.132	0.149	0.752
HG32	170	330	580	730	520	0.68	20	450	4.8	46	1200	2000	680	920	0.208	0.185	1.338
HG42	670	510	500	700	2200	150	480	1600	7.4	95	1600	15,000	1400	2000	0.301	0.329	1.609
HG43	270	210	640	580	770	14	25	1800	440	4	780	4600	1800	920	0.755	0.625	3.491
HG74	220	200	600	520	360	13	130	2100	55	120	1400	2700	1300	1200	0.148	0.256	1.106
HG93	430	320	450	691	350	43	270	2200	75	49	2800	2800	1800	3500	0.292	0.371	1.935
HK23	380	380	700	960	1100	24	160	2100	110	140	2100	6800	780	2500	0.094	0.130	0.607
HK27	300	450	730	1320	1320	31	180	480	460	130	3000	4910	1110	2230	0.273	0.467	2.228
HK31	580	540	770	1220	2160	8.1	130	1690	172	3.4	1660	8210	2010	3020	0.218	0.377	1.537
HK34	500	460	890	1000	1500	39	220	1900	45	100	6000	5200	1100	2700	0.209	0.305	1.359

^a^ Total CYP in pmol CYP/mg protein; ^b^ POR activity of cytochrome c reductase (pmol/mg protein); ^c^ Cyt b_5_ was determined spectrophotometrically (pmol/mg protein); ^d^ CYP- and FMO-specific activity in pmol product/(mg protein × min): Phenacetin-O-deethylase (CYP1A2), Coumarine-7-hydroxylase (CYP2A6), (S)-Mephenytoin-N-demethylase (CYP2B6), Paclitaxel-6α-hydroxylase (CYP2C8), Diclofenac-4′-hydroxylase (CYP2C9), (S)-Mephenytoin-4′-hydroxylase (CYP2C19), Buferalol-1′-hydroxylase (CYP2D6), Chlorzoxazone-6-hydroxylase (CYP2E1), Testosterone-6β-hydroxylase (CYP3A4), Lauric acid-12-hydroxylase (CYP4A11), Methyl-p-tolyl sulfide oxidase (FMO); ^e^ Amounts of cabozantinib metabolites as peak area/incubation time.

**Table 2:** Correlation coefficient (r) among CYP- and FMO-linked catalytic activity and amounts of cabozantinib metabolites formed in microsomes.

	Total CYPs	CYP1A2	CYP2A6	CYP2B6	CYP2C8	CYP2C9	CYP2C19	CYP2D6	CYP2E1	CYP3A4	CYP4A11	FMO
M1	0.705 *	0.104	0.607 *	0.886 ***	0.785 **	0.142	−0.119	0.028	−0.065	0.918 ***	0.163	0.137
M2	0.794 **	0.280	0.841 ***	0.695 *	0.654 *	0.086	0.033	−0.015	0.140	0.935 ***	0.193	0.315
M3	0.811 **	0.216	0.728 **	0.776 **	0.737 **	0.130	−0.021	−0.043	0.030	0.947 ***	0.223	0.341

* *p* < 0.05; ** *p* < 0.01; *** *p* < 0.001.

**Table 3:** The characteristics of the kinetics of cabozantinib oxidation by CYP3A4 and this enzyme in the presence of cyt b_5_.

Enzyme	Metabolite	Kinetic Characteristics ^a^		
		V_Max_ (peak area/min/nmol CYP)	K_0.5_ (μM)	K_i_ (μM)
CYP3A4	Monohydroxy cabozantinib	1.50 ± 0.05	8.98 ± 1.04	947.87 ± 202.96
	Desmethyl cabozantinib	1.44 ± 0.03	11.69 ± 1.00	NA
	Cabozantinib *N*-oxide	1.54 ± 0.04	9.56 ± 0.69	NA
CYP3A4 + cyt b_5_	Monohydroxy cabozantinib	3.60 ± 0.11	26.00 ± 2.92	NA
	Desmethyl cabozantinib	5.70 ± 0.24	38.53 ± 5.35	NA
	Cabozantinib *N*-oxide	9.96 ± 0.92	32.03 ± 5.68	490.22 ± 139.7

^a^ Values represent means ± SD of three independent in vitro incubations (*n* = 3). NA—not applicable.

## Abbreviations

α-NF	α-Naphthoflavone
AO	Aldehyde oxidase
CYP	Cytochrome P450
Cyt b_5_	Cytochrome b_5_
DDTC	Diethyldithiocarbamate
FMO	Flavin-containing mono-oxygenase
HPLC	High-performance liquid chromatography
M_1-3_	Cabozantinib metabolites
MET	Hepatocyte growth factor receptor
POR	NADPH:CYP oxidoreductase
RET	Rearranged during transfection receptor
TKI	Tyrosine kinase inhibitor
VEGFR	Vascular endothelial growth factor receptor
